# Increased Expression of 9-*Cis*-Epoxycarotenoid Dioxygenase, *PtNCED1*, Associated With Inhibited Seed Germination in a Terrestrial Orchid, *Phaius tankervilliae*

**DOI:** 10.3389/fpls.2018.01043

**Published:** 2018-07-17

**Authors:** Yung-I. Lee, Ming-Chuan Chen, Li Lin, Mei-Chu Chung, Wei-Ming Leu

**Affiliations:** ^1^Department of Biology, National Museum of Natural Science, Taichung, Taiwan; ^2^Department of Life Sciences, National Chung Hsing University, Taichung, Taiwan; ^3^Institute of Biotechnology, National Chung Hsing University, Taichung, Taiwan; ^4^Institute of Plant and Microbial Biology, Academia Sinica, Taipei, Taiwan; ^5^Advanced Plant Biotechnology Center, National Chung Hsing University, Taichung, Taiwan

**Keywords:** abscisic acid, orchid, seed development, 9-*cis*-epoxycarotenoid dioxygenase, germination

## Abstract

The phytohormone abscisic acid (ABA) is involved in regulating seed dormancy and germination. A crucial step of ABA biosynthesis in higher plants is the oxidative cleavage of *cis*-epoxycarotenoids by 9-*cis*-epoxycarotenoid dioxygenase (NCED). Seed development in orchids is unusual because the embryos are minute in size, without obvious histodifferentiation, and lack endosperm. To understand the regulation of ABA biosynthesis in orchid seeds, we isolated and characterized a full-length cDNA encoding an NCED homolog, *PtNCED1*, from developing seeds of an ornamental orchid, *Phaius tankervilliae*. Germination percentage was high at 90 days after pollination (DAP), when a globular embryo proper with a degenerating suspensor was evident. After 90 DAP, seed maturation was accompanied by a decrease in water content and a concomitant increase in ABA content and *PtNCED1* mRNA level along with a marked decrease in germination percentage. Mature seeds pretreated with NaOCl solution lowered ABA content and improved seed germination. Moreover, after seed germination, developing protocorms could respond to dehydration stress. Dehydration of protocorms stimulated an increase in *PtNCED1* level along with ABA content. Our results provide evidence of the involvement of *PtNCED1* in regulating endogenous ABA content in developing seeds and protocorms. The accumulation of endogenous ABA content in orchid seeds may have a critical role in seed dormancy and the protocorm response to water stress after seed germination.

## Introduction

Orchids produce numerous tiny seeds within a capsule. At the time of capsule maturity, an orchid seed contains an undifferentiated globular-shaped embryo covered by a thin testa without endosperm (Arditti and Ghani, 2000). In the natural environment, mycorrhizal fungi are required for successful germination and establishment of seedlings (Rasmussen, 1995), whereas in asymbiotic cultures in the absence of mycorrhizal fungi, the addition of soluble sugars and mineral nutrients are needed for germination and seedling establishment (Knudson, 1922). After germination, orchid embryos first show a temporary tubercle structure called a protocorm from which a shoot and a root differentiate to form a seedling (Yeung, 2017).

*Phaius tankervilliae* is a terrestrial species, known as “nun” orchid, and is native to the lowland forest in tropical Asia. It is a popular ornamental orchid used as a potted plant or garden plant (Moir, 1983). In general, seeds of terrestrial orchids are considered more difficult to germinate than those of epiphytic orchids (Arditti et al., 1982). The reasons for the poor germination may relate to the impermeable seed coat (Veyret, 1969; Lee et al., 2005) and/or the accumulation in mature seeds of inhibitory substances, such as phenolics (Kako, 1976) and abscisic acid (ABA) (Van Waes and Debergh, 1986; Van der Kinderen, 1987). NaOCl solution treatment of mature seeds significantly improved germination percentage, which indicates the removal of germination inhibitors (Van Waes and Debergh, 1986; Lee et al., 2007). In a recent study, mature seeds of *Cypripedium formosanum* showed high endogenous ABA content, and reducing the ABA content by fluridone application improved seed germination (Lee et al., 2015). These results indicate that ABA has a regulatory role in embryo development and seed dormancy of orchids.

The phytohormone ABA plays a crucial role in regulating the signaling networks associated with adaptation to stressful environments and physiological processes including stomatal movement and seed dormancy (Nambara and Marion-Poll, 2005). In higher plants, enzymes involved in biosynthesis of ABA and the pivotal role of ABA during transition from seed maturation to germination has been studied in great detail (Yan and Chen, 2017). ABA is *de novo* synthesized from carotenoids in plastids. The oxidative cleavage of 9-*cis*-epoxycarotenoids by the 9-*cis*-epoxycarotenoid dioxygenase (NCED) to produce xanthoxin is the first committed and also the rate-limiting step for ABA biosynthesis. Xanthoxin is subsequently exported from the plastids to cytosol and be converted to ABA via abscisic aldehyde (Nambara and Marion-Poll, 2005). In *Arabidopsis*, because the enzymes acting before or after NCED are all encoded by a single locus, the five-membered NCED gene family may play a spatio-temporal regulatory role in ABA biosynthesis (Iuchi et al., 2001; Tan et al., 2003; Frey et al., 2012).

In *Arabidopsis*, *AtNCED6* is expressed specifically in endosperm and *AtNCED9* in both embryo and endosperm. Genetic analysis of mutants suggested that *AtNCED6* and *AtNCED9* are essential for inducing and maintaining seed dormancy (Lefebvre et al., 2006). However, little is known about the roles of NCEDs for ABA biosynthetic activities in seed germination and protocorm development of orchids. To date, only a quantitative proteomic study of the symbiotic germination of *Oncidium* orchid detected an induced accumulation of NCED proteins, thus suggesting that ABA may promote the development of arbuscules in root cortical cells (Valadares et al., 2014). The basic knowledge of seed physiology in terrestrial orchids would benefit mass propagation to meet commercial needs in ornamental plant markets.

To gain further understanding of the role and regulation of ABA during seed development and germination in *P. tankervilliae*, we cloned and characterized a new gene encoding an NCED homolog, *PtNCED1*. In this study, we aimed to (1) analyze and characterize *PtNCED1* from *P. tankervilliae* seeds, (2) investigate the associations among water content, ABA content and *PtNCED1* transcript level and seed germination percentage, (3) investigate the effect of pretreating seeds with NaOCl solution on ABA content and seed germination, and (4) investigate the effect of dehydration treatment of protocorms on *PtNCED1* and ABA levels. The results demonstrate that the expression of *PtNCED1* plays an important role in regulating ABA content in orchid seed and protocorm development.

## Materials and Methods

### Plant Material

The plants of *P. tankervilliae* were cultivated in a greenhouse at the National Museum of Natural Science, Taichung, Taiwan. The flowers were manually pollinated at anthesis in March. In each experiment, capsules were randomly collected at regular intervals of 15 days from 45 to 120 days after pollination (DAP).

### Histology

The developing seeds were fixed in a solution of 2.5% glutaraldehyde and 1.6% paraformaldehyde in 0.1 M phosphate buffer (pH 6.8) for 4 h at room temperature. After fixation, the samples were dehydrated with an ethanol series and embedded in Technovit 7100 (Kulzer & Co., Germany) as described (Yeung and Chan, 2015). Sections of 3-μm thick were cut with glass knives by using a Reichert-Jung 2040 Autocut rotary microtome. Sections were stained with periodic acid-Schiff (PAS) reaction for total insoluble carbohydrates, and counterstained with 0.05% (w/v) toluidine blue O in benzoate buffer for general histology or 1% (w/v) amido black 10B in 7% acetic acid for protein (Yeung, 1984). Sections were examined and captured digitally by using a CCD camera attached to a light microscope (Axio Imager A1, Carl Zeiss AG). More than 100 capsules were used for the study of seed development and more than 600 different protocorms of each developmental stage were observed.

### Cloning of *PtNCED1* From *P. tankervilliae*

An RNA-Seq dataset was generated from developing seeds (90 DAP) of *P. tankervilliae* on an Illumina Hiseq 2000 platform. The raw data were cleaned by removing reads containing adapter and low-quality reads, yielding c. 53 million cleaned reads (i.e., ∼26 M paired-end reads, read length 100 bp). FASTQ sequence files were deposited at the National Center for Biotechnology Information (NCBI) under BioProject PRJNA445551 and sequence read archive (SRA) database SRP139691. The clean reads were assembled by using the Trinity method (Grabherr et al., 2011) with optimized *k*-mer length of 25 into 45688 contigs and deposited in the Transcriptome Shotgun Assembly (TSA) database at the NCBI under accession number GGMF01000000. We used the AtNCED6 and 9 protein sequences (encoded by AT3G24220 and AT1G78390, respectively) as queries to perform tBLASTn searches among the RNA-Seq dataset. Only one contig (Contig1245, or GGMF01001196 in the TSA database) was found to encode an NCED-like sequence. We named it as *PtNCED1* and deposited it in the NCBI with accession number MH105783 (see details in “Results” section).

### Southern Hybridization Analysis

About 100 μg genomic DNA was isolated from approximately 1.5 g seeds of *P. tankervilliae* by the CTAB method. For each enzyme digestion, approximately 20 μg genomic DNA was used. Samples were resolved on 1% agarose gel, blotted onto a nylon membrane, then probed with a ^32^P-labeled *PtNCED1* cDNA fragment. The cDNA fragment was PCR-amplified by using NCED-8, 9 primers (Supplementary Table S1) with p32H-NCED (see below) as a template. Isotope labeling was performed according to Amersham Megaprime DNA labeling systems (GE Healthcare Life Sciences, Marlborough, MA, United States). Hybridization and probe stripping were performed as described (Wang et al., 2011).

### Phylogenetic Analyses

Sequences of all NCED/CCD members in the genome of *Arabidopsis* and rice were retrieved from TAIR^1^ and RGAP^2^, respectively. Other representative members of NCED/CCD from various species were obtained from the NCBI. Multiple alignments of amino acid sequences involved use of the NCBI Constraint-based Multiple Alignment Tool (COBALT^3^, Papadopoulos and Agarwala, 2007). The phylogenetic tree was constructed by the Neighbor-Joining method with protein distance estimated by the Grishin correction method.

### Transient Overexpression of *PtNCED1* in Tobacco Leaves

For overexpressing PtNCED1 in plant cells, the *PtNCED1* cDNA fragment was amplified by RT-PCR with NCED-8, 9 primers (Supplementary Table S1), digested by PstI and BamHI restriction endonucleases, then subcloned into the pEpyon32H binary vector (C.-H. Yang, unpublished data) to produce the p32H-NCED plasmid. The pEpyon32H vector contains the mGFP5 reporter gene (Haseloff, 1999) under control of a 2x35S CaMV promoter. For overexpressing the PtNCED1-mGFP5 fusion protein (abbreviated as PtNCED1-GFP), a plasmid similar to p32H-NCED was constructed but using a primer that does not contain a stop codon (NCED-10, Supplementary Table S1) instead of the NCED-9 primer. After sequencing confirmation, both plasmids were transformed into *Agrobacterium* strain C58C1. Agroinfiltration was performed according to Cheng et al. (2010) on leaves of 4-week-old *Nicotiana benthamiana*. After transient expression for 3 days under a 16/8-h photoperiod at 25 ± 2°C, the subcellular distribution of PtNCED1-GFP was visualized by confocal microscopy (Olympus, IX81; Software, FV1000). Wavelengths of excitation/emission for chlorophyll and GFP were 470/633 and 488/510 nm, respectively. Furthermore, content of endogenous ABA was measured in agroinfiltrated tobacco leaves as described below.

### Real-Time PCR

Total RNA was extracted from developing seeds by using the RNeasy Plant Mini Kit (Qiagen, Hilden, Germany) according to the manufacturer’s instructions. RNA samples were treated with RQ1 DNase (Invitrogen, United States) to remove DNA remnants, then synthesis of the first cDNA strand involved using the PrimeScript RT reagent Kit (TaKaRa Bio, Japan). The *Ptactin-7-like* gene (Contig62, or GGMF01000062 in the TSA database, also deposited in the NCBI with accession number MH124736) was used as an internal quantification standard. Primers for real-time PCR were designed by using Premier 5.0 (Premier Biosoft, India, Supplementary Table S1) and each real-time PCR experiment involved 7.5 μL of SYBR Premix Ex Taq II (TaKaRa Bio), 1.5 μL cDNA, and 0.3 μL primers, and water was added to 15 μL. Each sample was analyzed in three biological replicates with three technical replicates by using the LightCycler 480 II Real-Time PCR System (Roche, Switzerland) with its relative quantification program. The parameters of reactions were an initial denaturation at 95°C for 30 s, then 40 cycles of 95°C for 5 s, and 60°C for 30 s. The 2^-ΔΔ*C*_t_^ method was used for evaluating gene expression.

### *In Vitro* Germination

After surface sterilization, seeds were removed from capsules and placed onto 20 ml culture medium in a 9-cm diameter Petri dish. The culture medium for seed germination is the modified Murashige and Skoog (MS) medium (Murashige and Skoog, 1962) which contained 1/4 strength of macroelements with full strength microelements (2 mg glycine, 0.5 mg niacin, 0.5 mg pyridoxine HCl, 0.1 mg thiamine, 100 mg myo-inositol, 20 g sucrose and 6 g agar per liter). The pH was adjusted to 5.7 before autoclaving at 102 kPa and 121°C for 20 min. The cultures were maintained in the growth room under light (20 μmolm^-2^ s^-1^) with a 12/12-h photoperiod at 25 ± 2°C. Experiments were performed in a randomized design and repeated three times. Twelve replicates (plates) were used for each treatment, with a minimum of 200 seeds per plate. Each plate was examined monthly by using a stereomicroscope (Carl Zeiss AG, Germany), and germination percentage was scored after 120 days of culture. Germination was defined as emergence of the embryo from the testa.

### Measurement of Water Content of Developing Seeds

Three capsules were randomly collected at intervals of 15 days from 45 to 120 DAP. Seeds of 0.1 g at different developmental stages were dissected carefully from the placenta and then dried at 70°C for 48 h. The water content was estimated as the percentage of water loss: fresh weight minus dry weight, to its fresh weight.

### Measurement of the Endogenous ABA Level

The procedure for endogenous ABA measurement was described in detail by Lee et al. (1993). In this study, samples for ABA measurement had three replicates, including seeds at each developing stage, or for seed pretreatments, protocorm for water stress experiments, and the agroinfiltrated tobacco leaves for transient overexpression of *PtNCED1*. In each replicate, seeds of 20 mg, protocorms of 100 mg and tobacco leaves of 700 mg were homogenized with mortar and pestle in a 1.5-mL Eppendorf tube containing the extraction solution (80% methanol and 2% glacial acetic acid). For estimating extraction efficiency, an internal standard, 166.5 Bq DL-[G-^3^H]-ABA (Amersham Biosciences, Buckinghamshire, United Kingdom) was added to the Eppendorf tube during the extraction procedure. Extraction was carried out at 4°C with shaking for 48 h in darkness. Extracts were filtered through filter paper (Whatman No. 1), and then further rinsed twice with extraction solution. The filtrates were dried in vacuo at 30°C then resuspended in 100% methanol. A solution of 0.2 M (NH_4_)_2_HPO_4_ was subsequently added and the samples were allowed to stand for 10 min at 4°C until ammonium salts formed. In order to remove pigments, phenolics and polar compounds, extracts were first passed through a polyvinylpyrrolidone (PVP) column, then a C18 cartridge (Waters, Milford, MA, United States). ABA trapped in the C18 cartridge was then eluted with 55% methanol. Eluates of 100 μl of each extraction were subjected to scintillation counting to determine ABA recovery, and the average recovery ranged from 70 to 76%. The eluates were dried in vacuo and resuspended in Tris-buffered saline (50 mM Tris-HCl, 10 mM NaCl, 1 mM MgCl_2_, 15mM NaN_3_, pH 7.5) and stored at -20°C for enzyme-linked immunosorbent assay (ELISA). ABA was quantified by ELISA according to Walker-Simmons (1987) using a Phytodetek^®^ ABA ELISA kit (Agdia, Elkhart, IN, United States). ABA levels are expressed as ng/mg fresh mass.

### Pretreatments With NaOCl Solution and the Application of Exogenous ABA on Seed Germination

For evaluating the effect of sodium hypochlorite solution, mature seeds at 120 DAP were collected and soaked in 0.5% NaOCl solution with two drops of a wetting agent Tween 20 (Sigma-Aldrich Co. St. Louis, MO, United States) for 10 or 20 min. For the control, seeds were soaked only in water. After treatments, seeds were rinsed three times with sterilized water and then placed on the culture medium or analyzed for ABA content. To examine the effect of exogenous ABA on seed germination, the mature seeds at 120 DAP which were pretreated with 0.5% NaOCl solution for 20 min were inoculated on the culture medium with the supplement of 0.0003, 0.0037, 0.0378, or 0.3787 μM (these concentrations were equal to 0.1, 1, 10, or 100 ng/mL) (±)-ABA (A1049, Sigma-Aldrich Co.). The culture medium without ABA supplement was used as control. ABA solution was filter-sterilized and added to the medium after autoclaved sterilization. The germination percentage was scored after 50 days of culture.

### Water Stress Experiments in Protocorms

Protocorms of about 2 mm in diameter were dehydrated by placing onto a 9-cm diameter Petri dish containing MS medium (as described above) supplemented with 10% polyethylene glycol (PEG) 6000 (Sigma-Aldrich Co.) for 24 and 48 h. Cultures were placed under light (20 μmolm^-2^ s^-1^) with a 12/12-h photoperiod at 25 ± 2°C. The control non-stressed protocorms were kept in the MS medium without the addition of PEG 6000 and under the same conditions as dehydrated protocorms for 24 and 48 h. Each sample was analyzed in three biological replicates and repeated three times. Each biological replicate (Petri dish) contained at least 30 protocorms.

### Statistical Analysis

Except for measurement of ABA content in the *PtNCED1* transiently expressed tobacco leaves in which an unpaired Student’s *t*-test was applied, all other statistical analyses were carried out with SAS statistical software version 8.2 (SAS Institute, Cary, NC, United States). The data were analyzed using analysis of variance (ANOVA) in combination with Fisher’s protected least significant difference test at *P* < 0.05. Data were arsine-transformed before performing ANOVA.

## Results

### Histological Observations of Seed Development

**FIGURE 1:**
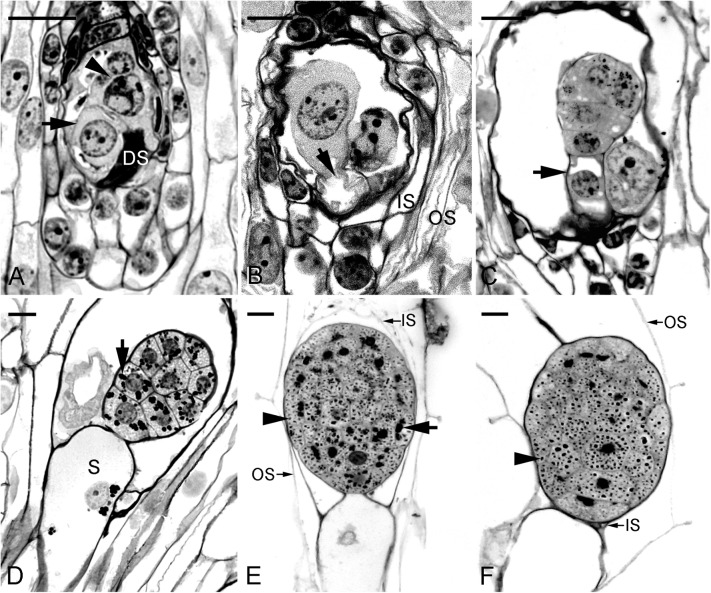
Light micrographs showing the embryo of *Phaius tankervilliae* at different stages of development. **(A)** A zygote (arrow) after fertilization at 40 days after pollination (DAP). In this species, the endosperm fails to develop, and the polar-chalazal complex (arrowhead) includes the chalazal nuclei and the polar nuclei. DS, degenerated synergid. **(B)** Elongated zygote at 45 DAP. Before the first cell division, the zygote is highly polarized with a nucleus located toward the chalazal end and a prominent vacuole (arrow) occupying the micropylar end. IS, the inner seed coat; OS, the outer seed coat. **(C)** Longitudinal section through a proembryo at 60 DAP. Vacuoles begin to enlarge at the suspensor cell (arrow). **(D)** Longitudinal section through a globular embryo with an enlarged single-celled suspensor (S) at 75 DAP. A number of starch grains (arrow) are present in the cells of the embryo proper. **(E)** Longitudinal section through a near-mature embryo at 90 DAP. At this stage, the suspensor has degenerated. Many starch grains (arrow) and protein bodies (arrowhead) are present in the cells of the embryo proper. The inner seed coat (IS) is dehydrating and compressing. **(F)** Longitudinal section through a mature embryo at 105 DAP. At this stage, the suspensor has degenerated. At maturity, the embryo is enveloped by the shriveled IS and the OS. Starch grains have disappeared and protein bodies (arrowhead) of various sizes are observed within the embryo proper. Although lipids could not be preserved in Historesin, plentiful translucent vesicles within the cytoplasm of embryo proper indicate the deposition of lipid bodies. Scale bar = 20 μm.

This study confirmed the earlier study by Ye et al. (1997) and documented clearly the time course of changes during embryo development. Fertilization occurred approximately 40 DAP (**Figure 1A**). From 45 to 60 DAP, the zygote and proembryo were observed within the capsules (**Figures 1B,C**). The zygote appeared as a highly polarized cell with a nucleus toward the chalazal end, and a prominent vacuole toward the micropylar end. Inside the endosperm cavity, the polar nuclei and one of the sperm nuclei formed the endosperm complex; these nuclei disintegrated rapidly as the embryo developed. However, endosperm was finally degenerated in the seed. In the early globular stage (75 DAP), the suspensor cell had enlarged with the process of vacuolation (**Figure 1D**). Within the developing embryo proper, starch granules began to accumulate and tended to congregate around the nucleus of the cell. At 90 DAP, the mitotic activity seemed to have ceased within the embryo proper and the suspensor started to degenerate (**Figure 1E**). At the same time, protein and lipid bodies were formed and accumulated within the embryo proper. At 105 DAP, the mature embryo filled with storage products was only six cells long and five to six cells wide without differentiation of apical meristem and cotyledon (**Figure 1F**). The seed coat was derived from the outer integument, which was only two cells thick. During early embryo development, the outer seed coat was highly vacuolated (**Figure 1A**). As the seed matured, a secondary wall was added to the radial and inner tangential walls of the outer layer. At maturity, the cells of seed coat became dehydrated and compressed into a thin layer (**Figure 1F**). Moreover, the dehydrated seed coat stained blue green with toluidine blue O, indicating the presence of phenolic compounds in the walls.

### Isolation and Characterization of *PtNCED1* From *P. tankervilliae*

To examine roles of ABA in seed and protocorm development in *P. tankervilliae*, we isolated a gene encoding NCED, the key enzyme for ABA biosynthesis in seeds. Because *AtNCED6* and *9* were known to be expressed predominantly during seed development and play key roles in regulating seed development and dormancy in *Arabidopsis* (Lefebvre et al., 2006), we looked for their homolog in the RNA-Seq dataset of *P. tankervilliae* by a tBLASTn search. Among 45688 assembled contigs, only one contig (Contig1245) was found to encode polypeptide that shares high sequence similarity with AtNCED6 or 9. The second similar subject found (Contig941, or GGMF01000904 in the TSA database) encodes polypeptide which share higher similarity to AtCCD1 than to AtNCED6 and 9, so was named as PtCCD1 in this study. Contig1245 contains a full-length open reading frame of 1830 nt with 257 and 487 nt as the 5′ and 3′-UTR, respectively. We named the deduced amino acid sequence as PtNCED1. PtNCED1 showed high sequence similarity (77–81%) with AtNCED2, 3, 5, and 9 but only 70% similarity with AtNCED6 (Supplementary Table S2).

A BLASTp search of the NCBI database revealed that PtNCED1 contains a highly conserved RPE65 domain for catalytic activity of the carotenoid cleavage dioxgenases (CCDs) and shares the highest sequence similarity (92%) with an NCED from *Dendrobium catenatum*. PSORT^4^ predicted a chloroplast stroma localization for PtNCED1. A transit peptide (1–53 amino acids of PtNCED1) was detected by ChloroP analysis (Emanuelsson et al., 1999). Sequence alignments for PtNCED1 with AtNCED6 and 9 together with the predicted transit peptide are in Supplementary Figure S1.

**FIGURE 2:**
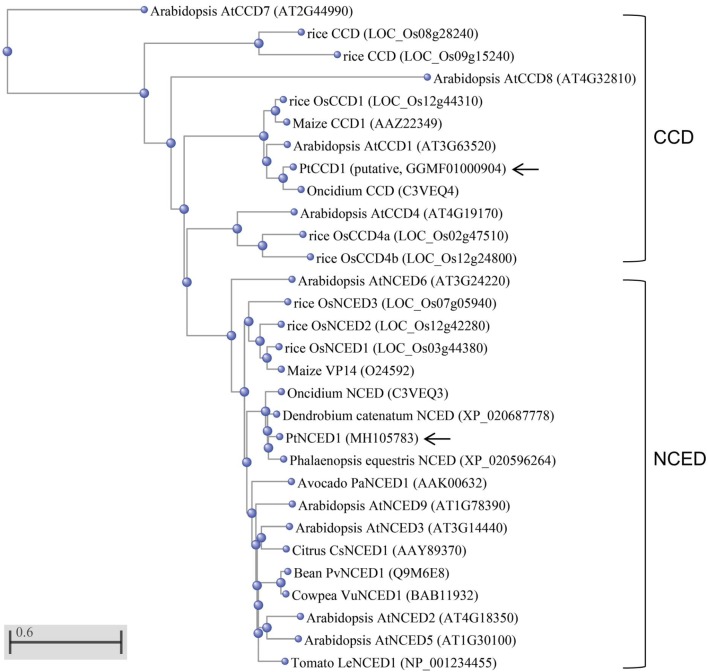
Phylogenetic analysis of PtNCED1 and other plant NCEDs and CCDs. The PtNCED1 protein sequence was aligned with the full-length NCED/CCD members from various species by using the COBALT program with Neighbor Joining as tree method in the NCBI. The scale bar indicates a genetic distance for 0.6 amino acid substitutions per site. Of note, members with very dissimilar sequences (>0.5) may only be accurate in grouping but not in genetic distance. Shown in bracket are the locus names for Arabidopsis and rice, obtained from TAIR (https://www.arabidopsis.org/) and MSU (http://rice.plantbiology.msu.edu/), respectively, and the accession numbers for other species, obtained from the NCBI. For rice, members of CCD and NCED follow the names given in Tan et al. (2003). PtNCED1 and PtCCD1 obtained in this study are denoted by arrows.

The NCED/CCD gene family has 8–9 members in both *Arabidopsis* and rice. To determine which member was most related to PtNCED1, we constructed a phylogenetic tree using the full-length protein sequences of NCED/CCD from various species. Located in the NCED clade, PtNCED1 shared the highest sequence similarity with NCEDs from orchids including *Oncidium* (Chiou et al., 2010), *D. catenatum*, and *Phalaenopsis equestris* (**Figure 2**). These orchid NCEDs are sister to NCEDs from rice and maize, then to a clade constituted by NCEDs from dicotyledon, and finally grouped with AtNCED6 to form the NCED clade. Of note, all NCED members are highly conserved (sharing >74% sequence similarity with each other) while sequences in most CCD members are quite divergent from each other. The putative PtCCD1 was most similar to the *Oncidium* CCD (Chiou et al., 2010) and grouped within the CCD clade.

**FIGURE 3:**
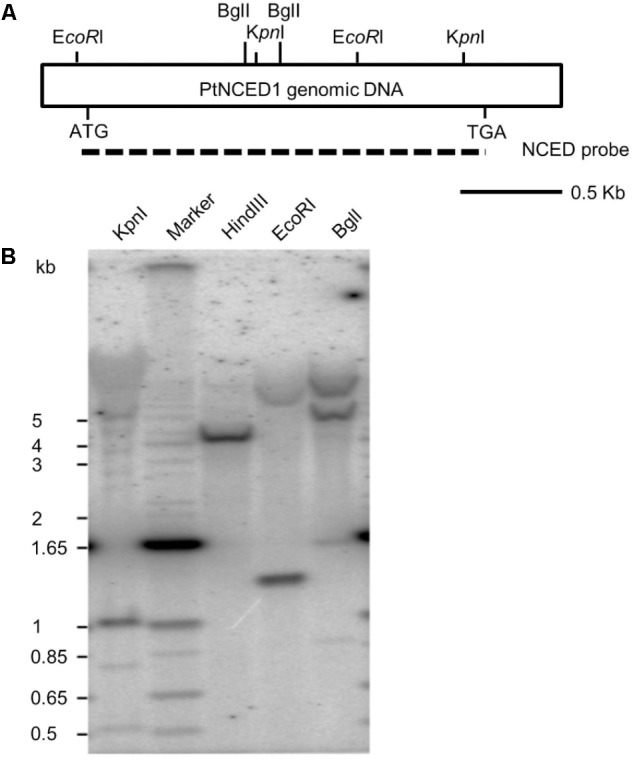
Southern hybridization of the *PtNCED1* gene. **(A)** Schematic depiction of the cDNA fragment of *PtNCED1* with *Eco*RI, *Bgl*I and *Kpn*I endonuclease sites shown. Dashed line indicates the open reading frame region used as a probe. **(B)** Southern blot analysis. Each lane was loaded with 20 μg genomic DNA from *P. tankervilliae* digested by the indicated restriction enzyme. The DNA markers are indicated on the left.

To examine the copy number of *PtNCED1* gene in *P. tankervilliae*, we performed genomic Southern analysis. Three restriction enzymes KpnI, EcoRI and BglI possessing cutting sites on the probe, each generated 2–3 prominent bands, and HindIII, which has no expected site on the probe region, showed only a single band (**Figure 3**). Because a high stringency wash (5% SDS, 65°C, then 1% SDS, 65°C) was carried out after hybridization, we concluded that no other genes sharing high sequence similarity with *PtNCED1* existed in *P. tankervilliae*. Nevertheless, some very faint bands detected in the KpnI and BglI-digested lanes still implicated the less-related NCED members. Moreover, a 1- and 1.3-kb band appeared in the KpnI- and EcoRI-digested lanes, respectively, which agrees with its length in the cDNA probe, therefore suggesting that *PtNCED1* is likely an intronless gene.

### Subcellular Localization of PtNCED1 in Chloroplasts

**FIGURE 4:**
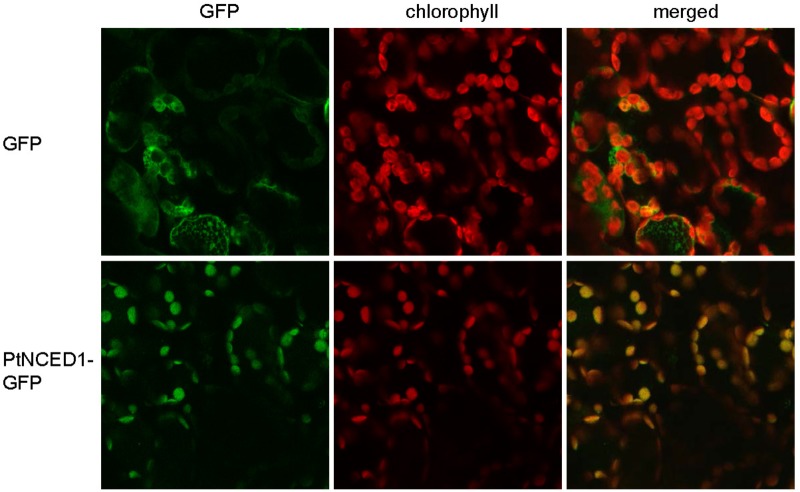
Subcellular localization of PtNCED1 in chloroplasts. *Nicotiana benthamiana* leaves were agroinfiltrated to express the GFP protein or the PtNCED1-GFP fusion protein for 3 days. The signal for GFP is shown in green, the autofluorescence of chlorophyll is red, and the co-localized signal is yellow.

Because NCED catalyzes the committed step for *de novo* synthesis of ABA, which occurs in plastids, we examined the subcellular localization of PtNCED1 by fusion with GFP and transient expression in tobacco leaves by agroinfiltration. On confocal microscopy, the free GFP proteins were distributed in the cytosol of mesophyll cells, whereas PtNCED1-GFP predominantly co-localized with chlorophyll in chloroplasts (**Figure 4**). Thus, PtNCED1 is mainly located in chloroplasts, most likely in the stroma as predicted by PSORT.

### Analysis of Enzyme Activity for PtNCED1

**FIGURE 5:**
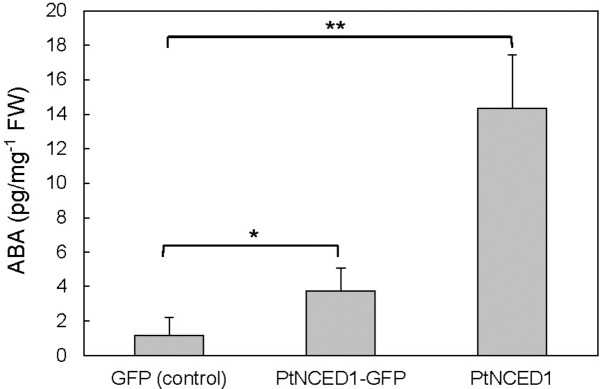
Abscisic acid content in the *PtNCED1* transiently expressed tobacco leaves. The materials were the same as in **Figure 4**. *N. benthamiana* leaves were agroinfiltrated to express the GFP, PtNCED1-GFP, or PtNCED1 protein for 3 days. Data are mean ± SD from three biological replicates. Significant difference was set at *P* ≤ 0.05 (^∗^) and *P* ≤ 0.01 (^∗∗^) by unpaired Student’s *t*-test.

To verify that PtNCED1 has NCED activity, we used agroinfiltrated tobacco leaves to overproduce PtNCED1 protein. ABA level was increased approximately 3- and 12-fold with overexpression of PtNCED1-GFP fusion protein and PtNCED1, respectively, as compared with the vector control (**Figure 5**). Therefore, PtNCED1 has NCED activity. The lower amounts of ABA produced by PtNCED1-GFP than PtNCED1 implies that GFP fusion may disturb the protein-folding and/or enzyme activity of PtNCED1 or, alternatively, decrease the protein abundancy.

### Effect of the Timing of Seed Collection on Germination

**FIGURE 6:**
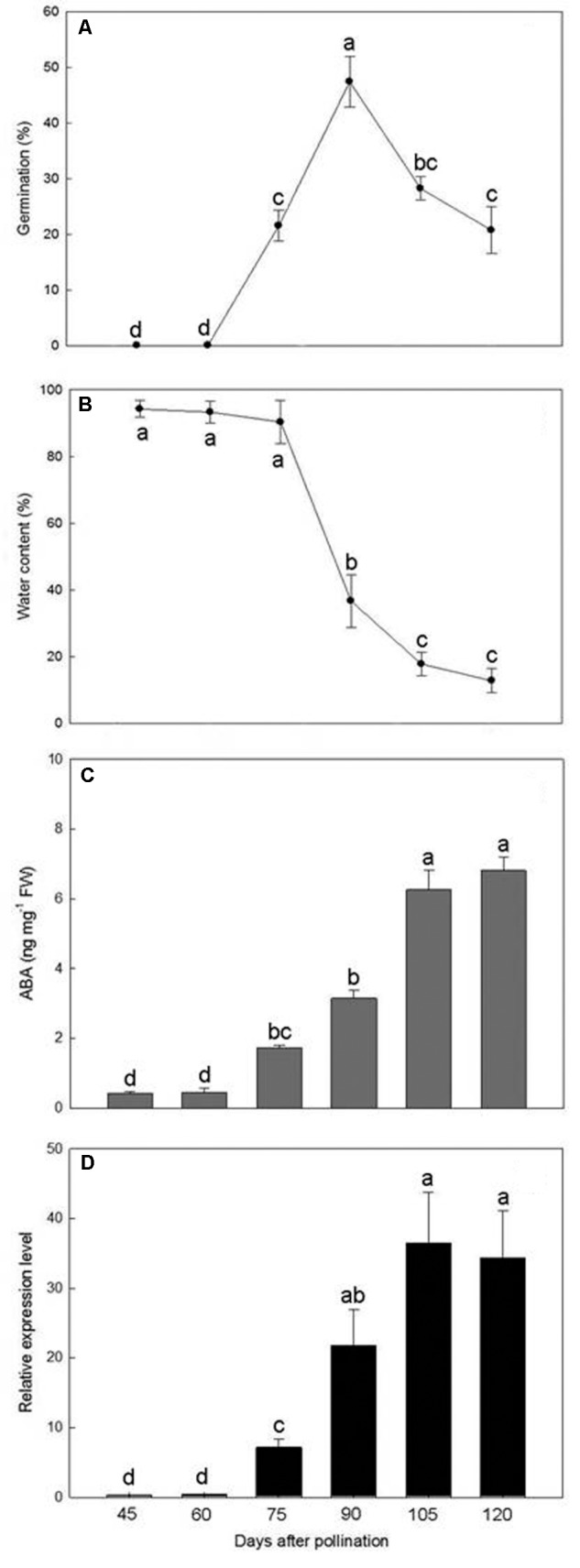
Changes in germination percentage **(A)**, water content **(B)**, ABA content **(C)**, and *PtNCED1* transcript level **(D)** in developing seeds. Data are mean ± SD from three biological replicates. Bars labeled with the same letter are not significantly different at *P* ≤ 0.05 by Fischer’s protected LSD test.

Germination was not observed in immature seeds at 45 and 60 DAP but thereafter increased to a maximum of 47.4% at 90 DAP (**Figure 6A**). However, as the seeds approached maturity, from 105 to 120 DAP, the germination percentage progressively decreased.

### Changes in Water Content in Developing Seeds

The water content of seeds collected from 45 to 75 DAP was more than 90% of the fresh weight (**Figure 6B**). During this period, the capsules were green and seeds were white and moist and still attached to the placenta. By 90 DAP, the seeds began to turn yellowish white with a sharp drop in water content. After 105 DAP, the seeds had turned yellow and became dry and free from the placenta, which allowed them to be readily shaken onto the medium. The water content of mature seeds (120 DAP) was estimated at 12.8%.

### Changes in Endogenous ABA Content and *PtNCED1* Level in Developing Seeds

During the early stages of seed development, from 45 to 60 DAP, the ABA content was maintained at low levels (0.43–0.44 ng/mg fresh weight) (**Figure 6C**). After 75 DAP, ABA content increased rapidly, with peak content (6.81 ng/mg fresh weight) at 120 DAP and continual increase from 105 to 120 DAP. Hence, the seeds of *P. tankervilliae* maintained a high ABA content for a prolonged period until the capsules split. Similar to changes in ABA content in developing seeds of *P. tankervilliae* (**Figure 6D**), the expression of *PtNCED1* was low during the early stage of seed development (45–60 DAP) but progressively increased from 70 DAP to peak at 105 DAP, then declined slightly when seeds matured.

### Effect of NaOCl Pretreatment and Exogenous ABA Application on Seed Germination

**FIGURE 7:**
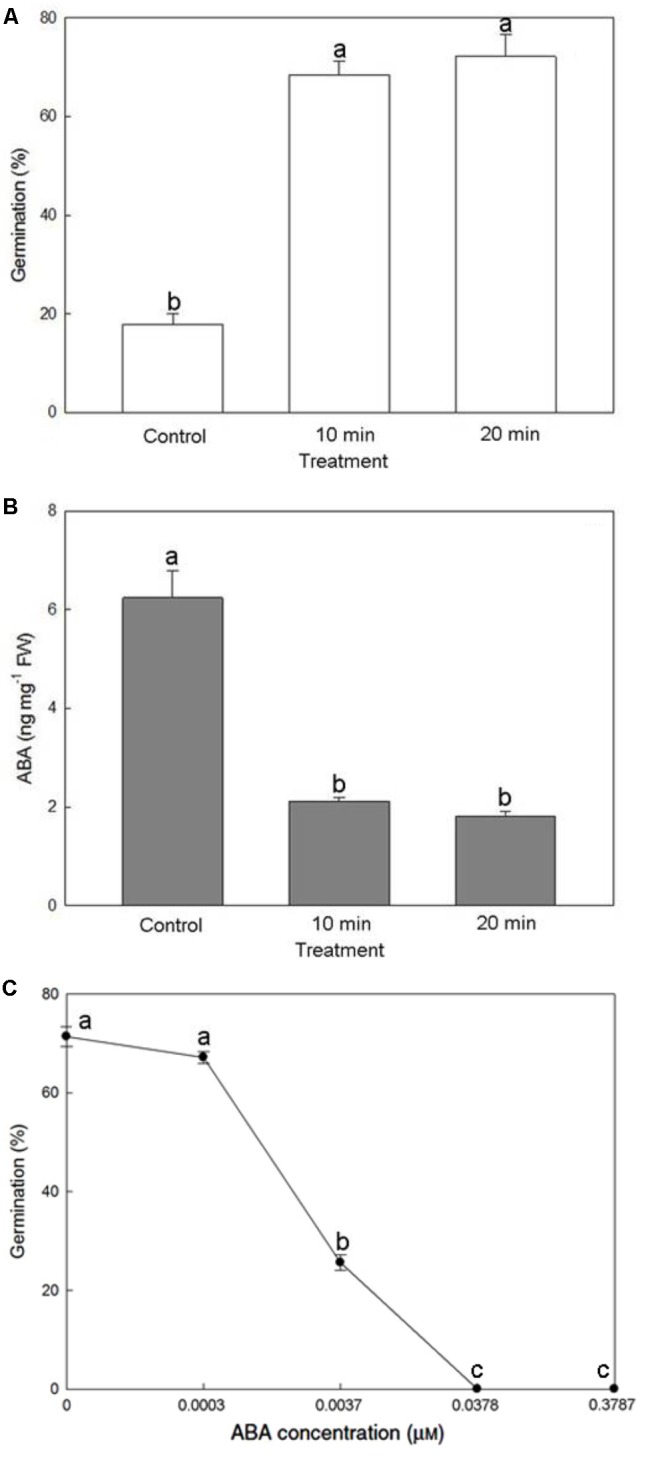
Effect of NaOCl pretreatment on seed germination **(A)** and endogenous ABA content within the mature seeds **(B)**, and effect of exogenous ABA application on seed germination after the NaOCl pretreatment **(C)**. Bars labeled with the same letter are not significantly different at *P* ≤ 0.05 by Fischer’s protected LSD test.

Soaking mature seeds with the 0.5% NaOCl solution for 10 or 20 min greatly increased seed germination percentage more than threefold, reaching >72% (**Figure 7A**). A substantial decrease of endogenous ABA content from 6.24 to 1.81 ng/mg fresh weight improved seed germination (**Figure 7B**), suggesting an effect by ABA removal. Moreover, applying a relatively low concentration of ABA (0.0037 μM, i.e., 1 ng/mL) in the culture medium was sufficient to suppress the germination of mature seeds pretreated with 0.5% NaOCl solution for 20 min (**Figure 7C**).

### Changes in *PtNCED1* Level and ABA Content in Response to Dehydration in Protocorms

**FIGURE 8:**
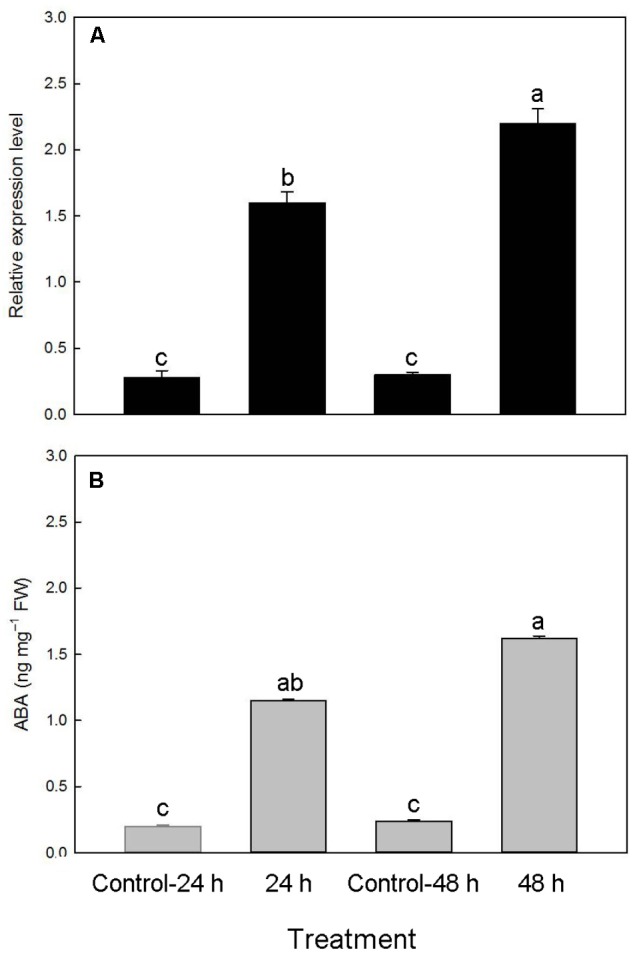
Changes in accumulation of *PtNCED1* transcripts **(A)** and ABA content **(B)** in response to dehydration in protocorms. 10% PEG 6000 was used for dehydration treatments for 24 and 48 h. Bars labeled with the same letter are not significantly different at *P* ≤ 0.05 by Fischer’s protected LSD test.

To examine whether the *PtNCED1* level could be induced by dehydration in protocorms, we quantified *PtNCED1* level in water-stressed protocorms and showed a 5.7- and 7.3-fold increase after dehydration for 24 and 48 h, respectively (**Figure 8A**). In agreement, ABA content increased from 0.2 to 1.15, then from 0.24 to 1.62 ng/mg fresh weight after dehydration (**Figure 8B**).

## Discussion

As consistent with our previous studies on other orchids (Lee et al., 2007, 2015), here we found that ABA accumulates during embryo development of *P. tankervilliae*, and we demonstrate for the first time that the key gene *PtNCED1* involved in ABA biosynthesis is indeed present in orchid. *PtNCED1* shares common characteristics with the known *AtNCEDs* and *OsNCEDs*. This work clearly indicates that orchids can synthesize ABA during embryogenesis and protocorm development.

### Characteristics of the Sole *PtNCED1* Transcript in Developing Seeds of *P. tankervilliae*

In *Arabidopsis*, each of *AtNCED* genes has its specific function — *AtNCED3* in abiotic stress response (Iuchi et al., 2001), *AtNCED5* in plant disease susceptibility (Fan et al., 2009), *AtNCED2* and *AtNCED3* in determining root growth direction (Tan et al., 2003), and *AtNCED6* and *AtNCED9* in seed development and dormancy (Lefebvre et al., 2006). Nevertheless, searching of the RNA-Seq dataset which we constructed from the developing seeds (90 DAP) of *P. tankervilliae* revealed only contig1245 encoding for a full-length NCED, together with other 6–7 contigs encoding for either full-length or truncated CCDs (data not shown). The lack of NCED isoforms may be due to the unique feature of seed development in orchids, e.g., the absence of endosperm and the rudimentary embryo (**Figure 1**). Moreover, as *AtNCED6* is expressed only in endosperm while *AtNCED9* in both embryo and endosperm (Lefebvre et al., 2006), it is not surprising that PtNCED1 is obviously closer to AtNCED9 than to AtNCED6 in phylogenetic analysis (**Figure 2**).

Although the two subfamilies NCED and CCD both possess the RPE65 domain and share significant protein sequence similarity with each other, their gene structures are very different. Most *CCD* genes contain multiple introns (5–13), whereas the *NCED* genes in *Arabidopsis* and rice are all intronless. Southern hybridization analysis confirmed that none intron existed in *PtNCED1* (**Figure 3**). To examine whether all sequenced *NCED* genes are intronless, we performed a tBLASTn search of the AtNCED6 protein sequence in the NCBI RefSeq Genome Database. Even by restricting the search targets to species distantly related to *Arabidopsis*, such as moss and algae, only intronless *NCED* genes were found, for example, NC_037277.1 from *Physcomitrella patens* (taxid:3218) and NC_009356.1 from *Ostreococcus lucimarinus* (taxid:242159).

In ABA biosynthesis, NCED is the key enzyme that cleaves an epoxycarotenoid precursor to form xanthoxin within plastids (Nambara and Marion-Poll, 2005). We examined the subcellular localization of PtNCED1 by fusion with a GFP protein tag. In accordance with the predicted transit peptide located on the N-terminal 1–53 amino acid region of PtNCED1 (Supplementary Figure S1), almost all PtNCED1-GFP signals were detected within chloroplasts (**Figure 4**). Moreover, examination of the transiently overexpressed PtNCED1 in agroinfiltrated tobacco leaves revealed a significant change in ABA content, approximately 3- and 12-fold increase for PtNCED1-GFP and non-fused PtNCED1, respectively (**Figure 5**). These analyses demonstrated the functionality of the cloned *PtNCED1* gene.

### Roles of *PtNCED1* and ABA Content in Developing Seeds of *P. tankervilliae*

In seed development, the spatio-temporal regulation of *NCED* genes is particularly important for control of ABA levels, which affect dormancy and germination (Nambara and Marion-Poll, 2005; Lefebvre et al., 2006). In this study, *PtNCED1* level continued to increase rapidly from 75 to 105 DAP and was maintained at this high level until the capsule split (120 DAP). This finding was associated with increased ABA content and decreased water content in developing seeds of *P. tankervilliae* (**Figure 6**). A similar change of ABA accumulation has been observed in other terrestrial orchids: *Calanthe tricarinata* (Lee et al., 2007) and *C. formosanum* (Lee et al., 2015). In response to desiccation as the seeds approaching maturity, dehydration and NCED upregulation usually act synergistically in enhancing ABA content in seeds (Lefebvre et al., 2006). ABA is involved in inducing the storage protein accumulation, the acquisition of desiccation tolerance and the regulation of seed dormancy (Karssen et al., 1983; Rivin and Grudt, 1991; McCarty, 1995; Kagaya et al., 2005). In most plants, ABA content usually peaks during the mid-stage of seed development, then declines as the seed approaches maturation (Goldbach and Michael, 1976; King, 1976; Kawakami et al., 1997). Different from most plants, orchid seeds maintain a high level of ABA at maturity (Van der Kinderen, 1987; Lee et al., 2007, 2015). Because the structure of orchid seed is simple, with a globular-like embryo housed within a thin seed coat and no endosperm, the embryo has little protection against seed desiccation as the seed matures. Maintaining a high ABA content may serve to protect the embryo under unfavorable germination conditions.

In developing seeds of *P. tankervilliae*, the endogenous ABA content keep increasing while approaching maturity. Pretreating mature seeds with 0.5% NaOCl solution for 10 and 20 min improved seed germination significantly to 72.2% and reduced endogenous ABA content (**Figures 7A,B**). Similar results of diminishing ABA contents in mature seeds have been observed in the pretreatment of hypochlorite solutions in *Dactylorhiza maculata* (Van Waes and Debergh, 1986) and *C. tricarinata* (Lee et al., 2007). Since NaOCl is an oxidizing agent, the endogenous ABA in seeds could be demolished through oxidation. In addition, different hypochlorites, e.g., Ca(OCl)_2_ and NaOCl have been used to scarify the seed coat that make the seed coat more hydrophilic and permeable (Lee, 2011; Barsberg et al., 2013). The increase in seed coat permeability may facilitate ABA leaching from mature seeds, and thus improved the germinability. In *C. formosanum*, the application of fluridone, an ABA synthesis inhibitor of developing seeds, reduced endogenous ABA content in mature seeds and improved the germination of mature seeds (Lee et al., 2015). In this study, the application of a small amount of exogenous ABA at 0.0037 μM was sufficient to inhibit the germination of immature seeds (**Figure 7C**), indicating that seed germination of *P. tankervilliae* is highly sensitive to a small amount of increase in ABA level. Together, these results confirm that ABA plays an important role in inducing and maintaining seed dormancy of orchids.

Upon seed germination, the orchid embryo first develops into a protocorm before forming a plantlet (Yeung, 2017). Under natural conditions, a protocorm without an active root system may be exposed to water stress under conditions of rainfall fluctuations. The *de novo* ABA biosynthesis induced by dehydration is an important avoidance/adaptation mechanism in response to stress (Lefebvre et al., 2006). In *Arabidopsis*, *AtNCED3* level has been shown to increase rapidly in response to dehydration, and transgenic plants overexpressing *AtNCED3* showed enhanced stress tolerance (Iuchi et al., 2001). In leaves of avocado and citrus, specific NCED isoforms, *PaNCED1* and *CsNCED1*, respectively, were required to adjust ABA content in response to drought stress (Chernys and Zeevaart, 2000; Rodrigo et al., 2006). In this study, we demonstrated that orchid protocorms can upregulate *PtNCED1* level, which leads to greater accumulation of ABA content in response to dehydration (**Figure 8**).

### *In Silico* Search of Genes Involved in ABA Biosynthesis and Catabolism in *P. tankervilliae*

In our TSA dataset, the *de novo* assembled ∼26M paired-end reads may contain a representative capture of the transcriptomes of developing seeds in *P. tankervilliae*. We were curious to have an overview for the gene members involved in ABA biosynthesis and catabolism. Therefore we used protein sequences of enzymes from *Arabidopsis* (Endo et al., 2014; Yan and Chen, 2017) as queries and performed tBLASTn against our RNA-Seq dataset in NCBI (TSA database, choosing *P. tankervilliae* as organism). For the ABA biosynthetic pathway, contigs encoding enzymes that shared 83, 78, 78, and 66% sequence similarity with ABA 1, 4, 2, and 3 were all found (Supplementary Table S3). However, contig corresponding the AAO3, an enzyme required for the last step conversion of *abscisic aldehyde* to ABA (Endo et al., 2014), was absent in our dataset. Instead, xanthine dehydrogenase (XDH) was revealed to share the highest sequence similarity (∼48%) with AAO3 (and also AAO1, 2, and 4). Although AAO3 and XDH both are molybdenum cofactor (MoCo)-containing enzymes and belong to the big oxidoreductase family, XDH has been characterized as a strict dehydrogenase but not an oxidase (Hesberg et al., 2004), therefore unlikely to substitute the role of AAOs. Interestingly, searching of the Transcriptomics Resource for the Orchid Family database (Orchidstra 2.0^5^) revealed that many orchids do not express AAO-like transcripts (data not shown). In the whole genome-sequenced orchid species, e.g., *P. equestris* (Cai et al., 2015) and *Apostasia shenzhenica* (Zhang et al., 2017), no AAO-like genes were found either. It would be interesting to examine whether minor routes, possibly via xanthoxic acid or abscisic alcohol as describe by Endo et al. (2014), may provide shunt pathways for ABA biosynthesis after the production of xanthoxin in orchids.

As the seed matured, ABA is converted into an inactive compound, 8′-OH-ABA, by CYP707A in most plants. In *Arabidopsis*, CYP707A1 and CYP707A2 play distinct roles for ABA degradation in the seed development and maturation/germination stages, respectively (Okamoto et al., 2006; Matilla et al., 2015; Yan and Chen, 2017). As two contigs encoding CYP707A-like proteins were found in our dataset (Supplementary Table S3), it would be worthy to investigate their gene expression profiles during seed development of orchids in future studies.

From the *in silico* analysis, we found an analogy between *P. tankervilliae* and other plants in the ABA metabolic pathway. Particularly, it is noted that the contig encoding *PtNCED1* possess an extremely high raw reads (16069) compared with other contigs (808–5074). This data indicates an abundancy of the *PtNCED1* transcripts in the developing seeds of *P. tankervilliae*, again implicates its critical roles there.

## Conclusion

In a terrestrial orchid, *P. tankervilliae*, we identified and characterized a 9-*cis*-epoxycarotenoid dioxygenase gene, *PtNCED1*. *PtNCED1* gene expression was increased in developing seeds and in water-stressed protocorms in a pattern consistent with the accumulation of ABA content. Pretreatments for improving germination of mature seeds could lower ABA content in seeds. These data suggest that *PtNCED1* is directly involved in ABA content regulation in seed dormancy and water stress responses in orchid protocorms.

## Author Contributions

Y-IL, Me-CC, and W-ML conceived the study. Y-IL and W-ML designed the study. LL and Mi-CC performed the molecular experiments. Y-IL performed histological study and hormone measurement experiments. Y-IL, W-ML, and Me-CC wrote the paper. All authors read and approved the final manuscript.

## Conflict of Interest Statement

The authors declare that the research was conducted in the absence of any commercial or financial relationships that could be construed as a potential conflict of interest. The reviewer KM and handling Editor declared their shared affiliation.

## Footnotes

1 https://www.arabidopsis.org/

2 http://rice.plantbiology.msu.edu/

3 https://www.ncbi.nlm.nih.gov/tools/cobalt/re_cobalt.cgi

4 https://psort.hgc.jp/

5 http://orchidstra2.abrc.sinica.edu.tw/orchidstra2/index.php
