# Supplementary material for: Increased Expression of 9-Cis-Epoxycarotenoid Dioxygenase, PtNCED1, Associated With Inhibited Seed Germination in a Terrestrial Orchid, Phaius tankervilliae
Source: Front Plant Sci. 2018 Jul 17;9:1043. doi: 10.3389/fpls.2018.01043 (PMC6056907; doi:10.3389/fpls.2018.01043)
Supplement: Supplementary file 1 [file Data_Sheet_1.PDF]

Supplementary Table S1. The primer pairs used in this study.

Unigene ID	Forward primer (5'-3')	Reverse primer (5'-3')
NCED-8/9 (with stop codon)	CACTGCAGATGGTCTCCTCCATGTCCTTG	CGGGATCCTCACTCCTGTGATTGGAGCTC
NCED-8/10 (no stop codon)	CACTGCAGATGGTCTCCTCCATGTCCTTG	CGGGATCCCTCCTGTGATTGGAGCTCACG
Ptactin 7-like gene for real-time PCR	ACAACAACGGCGGAACGG	GCTGCTCTTGGCGGTCTC
PtNCED for real-time PCR	GGTGTCGGGGTTCGCCAAG	CGACACGAGGTTGGAGGCG
