# Supplementary material for: Increased Expression of 9-Cis-Epoxycarotenoid Dioxygenase, PtNCED1, Associated With Inhibited Seed Germination in a Terrestrial Orchid, Phaius tankervilliae
Source: Front Plant Sci. 2018 Jul 17;9:1043. doi: 10.3389/fpls.2018.01043 (PMC6056907; doi:10.3389/fpls.2018.01043)
Supplement: Supplementary file 2 [file Data_Sheet_2.PDF]

Supplementary Table S2. Comparison of polypeptide sequences between PtNCED1 and AtNCEDs from *Arabidopsis*

	AtNCED2	AtNCED3	AtNCED5	AtNCED9	AtNCED6
identity (%)	67	70	65	70	54
similarity (%)	81	81	77	81	70
gap (%)	1	0	4	0	5

The full length protein sequences were aligned by using the BLAST2 program in the NCBI website.
