# Supplementary material for: Increased Expression of 9-Cis-Epoxycarotenoid Dioxygenase, PtNCED1, Associated With Inhibited Seed Germination in a Terrestrial Orchid, Phaius tankervilliae
Source: Front Plant Sci. 2018 Jul 17;9:1043. doi: 10.3389/fpls.2018.01043 (PMC6056907; doi:10.3389/fpls.2018.01043)
Supplement: Supplementary file 3 [file Data_Sheet_3.PDF]

Supplementary Table S3. Contigs in the Transcriptome Shotgun Assembly (TSA) database of *Phaius tankervilliae* that putatively encode proteins for ABA biosynthesis or degradation.

protein involved in ABA biosynthesis or degradation	corresponding gene in *Arabidopsis*	GenBank accession	protein sequence[a]	protein length (aa)	contig in TSA of *P. tankervilliae* [b]	GenBank accession[c]	contig length (nt)	putative protein sequence[a]	putative protein length (aa)	protein sequence identity (%)	protein sequence similarity (%)	protein sequence gap (%)	note	reads in SRA of *P. tankervilliae* [d]
zeaxanthin epoxidase, ZEP	ABA1	NP_851285.1	MGSTPFC	667	Contig7733	GGMF01007251	2593	MALFHGAL	661	71	83	0	full-length	2202
neoxanthin synthase, NSY	ABA4	NP_564889.1	MGFSSFIS	220	Contig5168	GGMF01004885	702	VQRSFLEC	184	55	78	0	ΔN-terminus	1694
9-cis-epoxycarotenoid dioxygenase, NCED	AtNCED6	NP_189064.1	MQHSLRSI	577	Contig1245	GGMF01001196	2554	MVSSMSLF	609	58	75	1	full-length	16069
xanthoxin dehydrogenase, XD	ABA2	NP_175644.1	MSTNTESS	285	Contig6195	GGMF01005832	1199	MSTADSSF	279	64	78	1	full-length	2082
molybdenum cofactor sulfurase, LOS5	ABA3	NP_564001.1	MEAFLKEF	819	Contig13014	GGMF01011886	1557	TGCFCNPC	411	50	66	3	ΔN-terminus	808
abscisic aldehyde oxidase, AAO	AAO3	NP_180283.1	MDLEFAVN	1332	Contig9315	GGMF01008684*	4519	MHIITVEGL	1270	31	48	10	full-length	2653
ABA 8'-hydroxylase 1 (CYP707A1)	AT4G19230	NP_974574.1	MDISALFLT	484	Contig6060	GGMF01005706	1550	MAISPILLIL	479	69	82	2	full-length	1652
					Contig2253	GGMF01002160*	1935	MEISYSLVY	475	61	77	1	full-length	5074
ABA 8'-hydroxylase 2 (CYP707A2)	AT2G29090	NP_180473.1	MQISSSSS	482	Contig6060	GGMF01005706	1550	MAISPILLIL	479	60	76	2	full-length	1652
					Contig2253	GGMF01002160*	1935	MEISYSLVY	475	57	72	0	full-length	5074

Indcated by grey background, the protein encoded by Contig9315 is not orthologous to AAO but to XDH (xanthine dehydrogenase).

[a,] only partial sequences are shown

[b,] by tBLASTn search against TSA dataset (Choose organism as Phaius tankervilleae, taxid:120015)
https://blast.ncbi.nlm.nih.gov/Blast.cgi?PROGRAM=tblastn&PAGE_TYPE=BlastSearch&BLAST_SPEC=&LINK_LOC=blasttab&LAST_PAGE=tblastn

[c], * denote that the protein is encoded by the reverse complement strand

[d,] by BLASTn search against Sequence Read Archive (SRA) dataset (Choose SRX3926271 and expand the max target sequences to 20000 in the Algorithm parameters.)
https://blast.ncbi.nlm.nih.gov/Blast.cgi?PROGRAM=blastn&PAGE_TYPE=BlastSearch&BLAST_SPEC=SRA&LINK_LOC=blasttab&LAST_PAGE=blastn
