# Supplementary material for: Increased Expression of 9-Cis-Epoxycarotenoid Dioxygenase, PtNCED1, Associated With Inhibited Seed Germination in a Terrestrial Orchid, Phaius tankervilliae
Source: Front Plant Sci. 2018 Jul 17;9:1043. doi: 10.3389/fpls.2018.01043 (PMC6056907; doi:10.3389/fpls.2018.01043)
Supplement: Supplementary file 4 [file Data_Sheet_4.PDF]

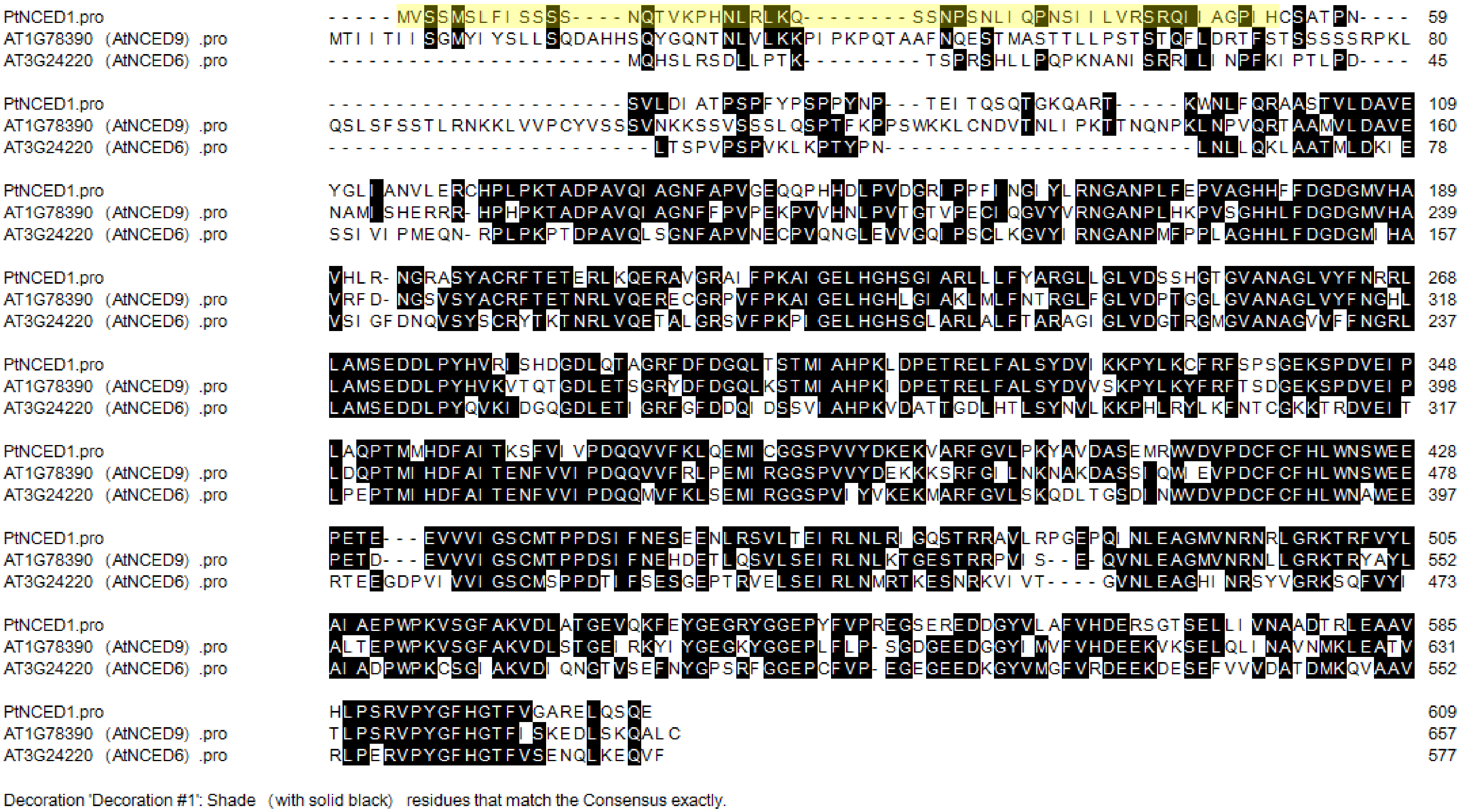

Supplementary Figure S1. Alignment of the deduced amino acid sequences of PtNCED1 with AtNCED6 and AtNCED9 from *Arabidopsis*. The black background denotes conserved residues. A dash indicates the missing amino acid. The transit peptide sequence is denoted with yellow background.
